# Nephrolithiasis: A Red Flag for Cardiovascular Risk

**DOI:** 10.3390/jcm11195512

**Published:** 2022-09-20

**Authors:** Alessia Gambaro, Gianmarco Lombardi, Chiara Caletti, Flavio Luciano Ribichini, Pietro Manuel Ferraro, Giovanni Gambaro

**Affiliations:** 1 Division of Cardiology, Department of Medicine, University of Verona, 37126 Verona, Italy; 2 Division of Nephrology, Department of Medicine, University of Verona, 37126 Verona, Italy; 3 U.O.S. Terapia Conservativa della Malattia Renale Cronica, Fondazione Policlinico Universitario A. Gemelli IRCCS, 00168 Rome, Italy

**Keywords:** calcification paradox, cardiovascular risk, ectopic calcification, hypertension, metabolic syndrome, nephrolithiasis, osteoporosis

## Abstract

Epidemiological evidence shows that nephrolithiasis is associated with cardiovascular (CV) morbidities. The association between nephrolithiasis and CV disease is not surprising because both diseases share conditions that facilitate their development. Metabolic conditions, encompassed in the definition of metabolic syndrome (MS), and habits that promote nephrolithiasis by altering urine composition also promote clinical manifestations of CV disease. By inducing oxidative stress, these conditions cause endothelial dysfunction and increased arterial stiffness, which are both well-known predictors of CV disease. Furthermore, the subtle systemic metabolic acidosis observed in stone formers with CV disease may have a pathogenic role by increasing bone turnover and leading to reduced mineral content and osteoporosis/osteopenia. Heart valves and/or coronary artery and aortic calcifications are frequently associated with reduced mineral density. This is known as the ‘calcification paradox’ in osteoporosis and has also been observed in subjects with calcium nephrolithiasis. Evidence supports the hypothesis that osteoporosis/osteopenia is an independent risk factor for the development of CV calcifications. In the long term, episodes of renal stones may occur from the onset of metabolic derangements/MS to arterial stiffness/atherosclerosis and CV morbidities. These episodes should be considered a warning sign of an ongoing and silent atherosclerotic process. The evaluation of cardiometabolic risk factors and MS components should be routine in the assessment of renal stone formers. This would allow for treatment and prevention of the development of CV complications, which are much more severe for the patient and for public health.

## 1. Introduction

The prevalence of kidney stones has increased worldwide in recent decades [1,2]. This is believed to be the consequence of the diffusion of the Western lifestyle and eating habits. Epidemiological research confirms this observation, as the prevalence of nephrolithiasis is higher in subjects with lifestyle-associated conditions, such as obesity [3], type 2 diabetes (DM2) [4], and metabolic syndrome (MS) [5,6,7].

Increased morbidity and mortality associated with nephrolithiasis was reported in the Global Burden of Disease study [8,9]. Furthermore, the health problems associated with acute episodes due to stone passage may seriously impair quality of life [10,11]. Nephrolithiasis is also associated with an increased risk of chronic kidney disease (CKD) and end-stage kidney disease (ESKD) [12], bone metabolic disease (BMD) and bone fractures [13,14], and cardiovascular (CV) disease [15]. The association between nephrolithiasis and CV risk factors has long been understood.

This review addresses the evidence supporting the association between nephrolithiasis and CV morbidities, the possible mechanism involved, and their clinical implications.

## 2. Nephrolithiasis and Cardiovascular Disease Are Associated: The Evidence

Epidemiological evidence that nephrolithiasis is associated with CV morbidities has been recently meta-analysed, taking into consideration prospective cohort studies in North America and Asia [15]. Coronary heart disease, myocardial infarction, and stroke were all significantly more frequent in subjects with previous renal stones than in subjects without renal stones, with a relative risk ranging from 1.21 to 1.24. Two other studies from Korea and Taiwan, not included in the meta-analysis, confirmed that nephrolithiasis was a significant risk factor for the development of stroke [16,17].

It is interesting that, in some of the studies, the risk of CV outcomes was higher in younger females [17,18,19,20], a population that is generally considered at low risk of developing CV disease.

In the studies that also evaluated the relationship between CV risk and the number of stone recurrences, the CV risk was proportionally higher [20].

Evidence of the association between nephrolithiasis and atherosclerosis was provided by the CARDIA study. Over 5000 subjects aged 18–30 years from the general population were followed for 20 years. In the 200 subjects who had symptomatic renal stones, the probability of having carotid stenosis or carotid intima-media thickness in the upper quartile at end of the follow-up period was 50–60% higher than in non-stone formers after adjustment for many potential confounders, from nephrolithiasis to vascular damage [21]. In a large cohort, nephrolithiasis assessed by kidney ultrasound was significantly associated with subclinical coronary artery calcification detected using cardiac tomography after adjustment for many CV and metabolic risk factors [22].

The association between nephrolithiasis and CV disease was not unexpected because both diseases share conditions that favour their development. The association between these conditions and nephrolithiasis will be reviewed.

## 3. The Associated Conditions

### 3.1. Smoking

Epidemiological studies have correlated increased smoking with increased prevalence of nephrolithiasis in the general population. In a large Taiwanese cohort, smoking was an independent risk factor for nephrolithiasis in males but not in females [23]. It has been proposed that smoking may increase oxidative stress in the kidney, which promotes lithogenesis [24].

As smoking is a relevant source of cadmium, the link between smoking and nephrolithiasis might be explained by the observation that higher levels of urinary cadmium were associated with increased calciuria [25] and a greater propensity for nephrolithiasis, notably in females [26]. However, the available evidence linking smoking to nephrolithiasis is scarce, inconsistent, and deserves further investigation.

### 3.2. Hypertension

A recent meta-analysis confirmed an increased risk of developing hypertension among stone formers [27]. In a general population cohort, the incidence of hypertension was significantly higher after the first episode of nephrolithiasis [28], after adjustment for several factors affecting the occurrence of hypertension. Despite some reports suggesting that hypertensive subjects are at increased risk of nephrolithiasis [29,30], studies on larger populations [31,32] and the meta-analysis [27] do not confirm this.

Studies on the general population generally lack sufficient information on stone composition. Most stones diagnosed in the general population are composed of monohydrated calcium oxalate. It is unclear whether less common stone compositions are associated with a specific risk of hypertension. With the limitation of a meta-analysis, Shang et al. observed that stone composition was not a predictor of incident hypertension [27].

It is unclear how the presence of kidney stones could lead to hypertension. Urine chemistry represents a possible link between the two conditions. Increased calciuria is frequent in nephrolithiasis and is also observed in some hypertensive subjects [33,34]. Familial aggregation of hypertension has been reported in patients with hypercalciuria [35] and increased calciuria has been observed in the offspring of hypertensive subjects [36]. Furthermore, hypertension is associated with tubular acidification defects and low urine citrate [37]. However, these urinary chemistry abnormalities are not convincing, as we should also expect that hypertension precedes the onset of nephrolithiasis, which is not supported by recent reports.

Nephrolithiasis is a well-known, although infrequent, cause of CKD and ESKD [12]; however, subtle renal parenchymal damage induced by nephrolithiasis is likely to be much more frequent.

Calcium oxalate crystals induce oxidative stress and increase the production of reactive oxygen species in tubular cells [38,39]. Urine biomarkers of tubular injury and chronic inflammation have been repeatedly described in renal stone patients [40,41]. Furthermore, the renal parenchyma is more frequently thinner in potential kidney donors who are stone formers vs. those who are not stone formers [42]. Finally, hypertension correlates with the extension of papillary calcifications [43]. These data suggest that frequent stone formers have elusive tubulointerstitial injuries that may ultimately manifest with hypertension.

Pathogenic factors beyond nephrolithiasis that play a role in increasing the risk of hypertension in stone formers cannot be ruled out. Genetics, habits, and environmental factors shared by both diseases could manifest early as nephrolithiasis and later as hypertension.

### 3.3. Diabetes Mellitus

The long-known association between DM2 and nephrolithiasis has been recently confirmed in a Bayesian meta-analysis [44]. Diabetes may both precede or follow the onset of nephrolithiasis [4,45]. Furthermore, impaired glucose tolerance/prediabetes is associated with nephrolithiasis [46,47]. The increased risk of developing renal stones in these conditions is likely secondary to insulin resistance [48]. Insulin resistance causes high plasma levels of free fatty acids, which impairs ammoniagenesis in the proximal tubule epithelium [49,50]. The consequent high acidity of the urine is a well-known risk factor for uric acid crystallogenesis and uric acid stones [51]. It was proposed that uric acid nephrolithiasis be added to the conditions of the clinical phenotype of insulin resistance [52].

Uric acid stones occur frequently in patients with insulin resistance (e.g., diabetes, obesity, MS) [52,53], although calcium oxalate stones are likely more frequent. This is explained by the observation that hyperglycaemia increases urinary calcium [54], phosphorous [54], uric acid [55], and oxalate [56].

### 3.4. Obesity

Studies have shown that high body mass index (BMI) [3,57] and several obesity-related indices [23] are associated with a higher risk of nephrolithiasis. Of particular interest are the results of a longitudinal study conducted on a large Taiwanese cohort that demonstrated an increased incidence of kidney stones in subjects with abdominal obesity [23]. Therefore, the type of obesity is important when explaining the risk of nephrolithiasis, as confirmed by a study in which total body fat and trunk fat were significantly associated with low urine pH and uric acid supersaturation [58].

Metabolically healthy obese subjects (about one-tenth of all the obese people) are at lower risk of developing symptomatic nephrolithiasis than unhealthy obese people, according to a study performed on a nationwide dataset of the Korean population [59]. This supports the idea that the association between obesity and nephrolithiasis is driven by insulin resistance. Moreover, compared to unhealthy obesity, metabolically healthy obesity is characterised by lower insulin resistance and visceral fat, a more satisfactory CV risk profile [60,61], and a lower risk of DM2, CV diseases, and death [62,63].

Insulin resistance, highly acidic urine [64], hypercalciuria [65], oxaluria [66], and uricosuria with possible heterogeneous nucleation [67] may favour calcium oxalate nephrolithiasis.

Furthermore, obesity may trigger oxidative stress and the production of reactive oxygen species in the kidney [68,69,70], which have been shown to promote lithogenesis [24].

### 3.5. Dyslipidaemia

A meta-analysis showed that dyslipidaemic subjects had a 1.6-fold higher risk of developing nephrolithiasis than non-dyslipidaemic subjects [44]. The hypothesis was that renal lipotoxicity was the pathogenic trigger of nephrolithiasis; i.e., lipid accumulation in proximal tubular cells induces the impairment of ammoniagenesis and acidic urine, thereby promoting uric acid lithiasis [50,71].

### 3.6. Metabolic Syndrome

MS encompasses several conditions that are associated with an elevated risk of atherosclerosis and CV disease [72]. Therefore, the increased CV risk observed in stone formers can be explained by the association of MS with nephrolithiasis.

Several studies and a meta-analysis established the association between nephrolithiasis and MS [3,5,6,7,44]. In stone formers, the risk of developing MS is almost double that of non-stone formers and even higher in patients with uric acid stones [73]. Moreover, the severity of nephrolithiasis is associated with the clustering of MS traits; the more traits simultaneously present, the more severe the history of the stone disease [74]. In parallel, stone composition exhibits a tendency toward uric acid stones with the clustering of MS traits [75,76,77].

As in diabetes, MS may both precede or follow the onset of nephrolithiasis [73,78].

Clustering of MS traits is associated with an increased likelihood of having high urinary excretion of calcium, uric acid, oxalate, and low excretion of citrate [74]. Furthermore, the number of MS traits is inversely associated with lower ammoniagenesis and urine pH [79], which are the likely consequence of insulin resistance [80]. All these urinary conditions increase the propensity for lithogenesis and may explain why subjects with MS are at an increased risk of becoming stone formers. A recent study by Hood et al. [77] highlighted the association between many urinary abnormalities and MS. In the study, performed on a large group of stone formers with MS, urine pH was the basic abnormality, while the other abnormalities were the consequence of higher dietary intake of protein.

A further possible mechanism is vascular dysfunction, an obvious consequence of MS, which may explain the association between MS and nephrolithiasis due to its promotion of the formation of Randall’s plaques [81,82].

However, these mechanisms do not account for the high risk of developing MS observed in stone formers [73]. The onset of the stone disease coincides with a generally evident clinical episode, while the definition of MS requires that certain thresholds be reached; however, the process of reaching these implies that the mechanisms causing MS are already active. Therefore, we speculate that a form of ‘pre-MS’ is already present at the first stone episode. Genetic findings support this conjecture. Two genome-wide association studies (GWAS) revealed the association between two variants of the gene GCKR and both MS [83] and nephrolithiasis [84,85]. GCKR encodes the liver glucokinase regulator protein [86]. The two variants are associated with high triglyceride levels and high fasting plasma glucose [87]; in addition to being a trait of MS, both are risk factors for nephrolithiasis. According to these observations, nephrolithiasis appears to be caused by the same metabolic derangements that cause MS.

## 4. The Logical Connection between Nephrolithiasis and CV Disease

**Figure 1:**
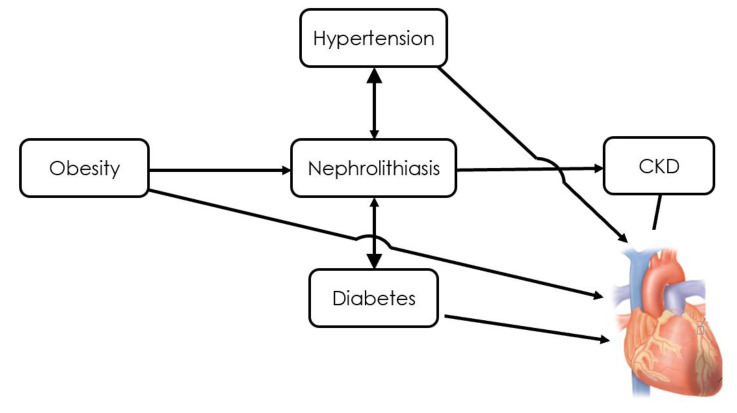
Nephrolithiasis and CV risk factors.

According to what has been previously discussed, there is extensive evidence explaining how several metabolic conditions, encompassed in the definition of MS, and habits that promote nephrolithiasis through alterations in urine composition lead to CV clinical manifestations (Figure 1).

A recent study demonstrated that vascular calcifications are highly prevalent in patients with nephrolithiasis, and that the associated stone phenotype is not related to uric acid levels but instead to the phosphate content of calcium stones [88]. However, well before the development of ectopic calcifications, the aforementioned conditions cause endothelial dysfunction, which likely represent the earliest pathogenic feature of CV disease [89]. Oxidative stress, which is shared in all features, plays a key role in the pathogenesis of endothelial dysfunction [89]. Interestingly, endothelial dysfunction has been demonstrated in stone formers [90,91]. This may contribute to the explanation of why idiopathic calcium stone formers have increased arterial stiffness [92], a known independent predictor of CV morbidity [93]. Interestingly, in secondary forms of calcium nephrolithiasis, arterial stiffness is not impaired [92], suggesting that this alteration is specific to the most common idiopathic calcium nephrolithiasis and that the epidemiological association with CV disease might be driven by these common features.

There might be more linking nephrolithiasis with CV disease. We observed that the association between incident stones and incident CV outcomes in three large prospective cohorts persisted after adjustments for multiple factors, including all known CV risk factors, drugs, and dietary intakes [18]. A similar residual association was observed by Kim et al. [22]. These observations indicate the contribution of still undefined conditions that cause both nephrolithiasis and CV disease.

Bargagli et al. investigated stone and urine composition in stone formers with and without CV morbidities; lower urinary excretion of citrate and magnesium were observed in the stone formers [94]. Hypocitraturia, which is common in stone formers [95], was associated with a higher prevalence of abdominal aortic calcification [96]. In a study of the general population, individuals with reduced magnesiuria had higher CV risk [97,98]. As citrate and magnesium play a role in the pathogenesis of atherosclerotic plaques and lithogenesis, these abnormalities might represent some of the undefined primary conditions that directly connect nephrolithiasis to CV disease [94].

Increased acid retention, and thus the existence a subtle systemic metabolic acidosis, was indicated in stone formers with CV disease [94]. This observation might be important when considering that, in stone formers with MS, the increased acid excretion is mainly the result of increased animal protein intake [77]. Therefore, we expect that in patients with a subtle derangement in systemic pH control, increased dietary protein intake induces a status of mild chronic acidaemia. Under such conditions, the bone buffers the excess acid, increasing bone turnover and leading to reduced mineral content and osteoporosis/osteopenia, which is further compounded by direct renal calcium leakage caused by the acid load [99].

## 5. Osteoporosis and the ‘Calcification Paradox’: The Missing Link between Nephrolithiasis and CV Disease?

Osteoporosis/osteopenia, which is frequently encountered in idiopathic calcium nephrolithiasis [100], may provide further insight in understanding the relationship between nephrolithiasis and CV disease. The alteration in bone metabolism, either as a primary condition or secondary to an excessive response to the buffering of the acid load generated by dietary proteins, likely has a pivotal role in inducing increased calciuria and lithogenesis [101]; however, it is also linked to a major risk of CV calcifications and mortality.

Vascular calcifications are strongly related to bone metabolism. Heart valves and/or coronary artery and aortic calcifications are frequently associated with reduced mineral density. This is known as the ‘calcification paradox’ of osteoporosis [102]. Interestingly, this paradox has also been observed in subjects with calcium nephrolithiasis [103].

Although osteoporosis and CV calcification share several cardiometabolic risk factors [104], consistent evidence supports the idea that osteoporosis/osteopenia is an independent risk factor for CV calcifications [105].

CV calcifications do not form from passive deposition of calcium, but via an osteogenesis-like phenomenon [106]. Therefore, it is likely that the paradox is caused by underlying biological factors that inversely connect the process of bone turnover with the formation of CV calcifications. Vitamin D is involved in both bone turnover and CV calcifications [107]. Furthermore, a genetic polymorphism of vitamin D shown to be associated with osteopenia was also associated with the risk of developing calcific aortic stenosis [107].

Gender variation has been observed in this phenomenon; the association between osteoporosis/osteopenia and aortic calcifications is stronger in females than in males [108,109,110]. This is interesting considering that the relationship between nephrolithiasis and CV disease seems to be stronger in women [111]. The gender difference could be explained by oestrogens, whose deficiency has a crucial role in favoring both osteoporosis and CV diseases in females. Furthermore, a specific polymorphism in an oestrogen receptor gene has been shown to be associated with both osteoporosis/osteopenia and valve calcifications in women [112,113,114]. Oestrogen deficiency has been suggested to explain why the incidence of nephrolithiasis in females, lower than in males, increases after both spontaneous and surgical menopause [115]. In view of these observations, it might be interesting to investigate whether females who develop renal stones at younger ages have oestrogen deficiency, which could explain a higher risk of CV disease and BMD.

## 6. Other Hypothesis Linking Nutrition, Nephrolithiasis and CV Disease

The intestinal microbiota is nowadays considered as an important metabolic organ that links the metabolism of nutrients to molecules that contribute to chronic diseases (hypertension, atherosclerosis, diabetes, chronic renal failure, etc.) [116]. A disruption of the normal gut microbiota has been observed in both patients with nephrolithiasis [117] and in patients with cardiovascular disease [116]. A possible link between the gut microbiota, nephrolithiasis, and CV disease is trimethylamine (TMA), the precursor of trimethylamine N-oxide (TMAO), a product of the gut microbiota metabolism of carnitine, choline and phosphatidylcholine. Interestingly, red meat is a strong contributor of TMA which is largely absorbed from the bowel into portal circulation, and oxidized to TMAO in the liver. TMAO was shown to accelerate atherosclerosis in a murine model, particularly in females who disclosed higher TMAO plasma levels [118]. In observational studies, TMAO plasma levels were strongly positively correlated with CV disease [119]. A recent study in a mice model of hyperoxaluria has revealed that TMAO aggravates hyperoxaluria-induced kidney injury enhancing autophagy, apoptosis and inflammation, and favoring calcium oxalate crystal deposition in renal tubular epithelia [120]. Thus, TMAO may be one of the missing links explaining the association between nephrolithiasis and CV disease.

Epigenetic modifications of genes involved in cellular and metabolic pathways shared by the tubular epithelium and the vessel wall may also contribute to the association between stone disease and CV morbidity. Nutrition is one of the environmental conditions that induces important epigenetic modifications. To our knowledge, the hypothesis that nutrigenomic modifications have a role in explaining the increased CV risk in stone formers has never been investigated, but we guess it should. In this regard, it is interesting to observe that fatty acids (FA), such as α-linolenic acid, eicosapentaenoic acid (EPA), and docosahexaenoic acid (DHA), modify DNA methylation sites in a number of genes associated with CV morbidities [121]. Nutritional factors can modulate the composition in FA of plasma and cell membrane phospholipids, and thus potentially exert epigenetic effects on a number of genes and metabolic pathways relevant to the pathogenesis of nephrolithiasis, atherosclerosis, and CV disease. Incidentally,, the manipulation of plasma and cell membrane phospholipid composition in a rat model and in renal stone patients changed urine ionic milieu creating conditions favoring crystallization, and also bone turnover [122,123,124]. Studies are necessary to confirm this hypothesis.

## 7. Practical Implications and Conclusions

The role of other mechanisms contributing to CV risk in nephrolithiasis remains under investigation. However, patients with nephrolithiasis frequently have several cardiometabolic risk factors that are often the consequence of diet and lifestyle.

**Figure 2:**
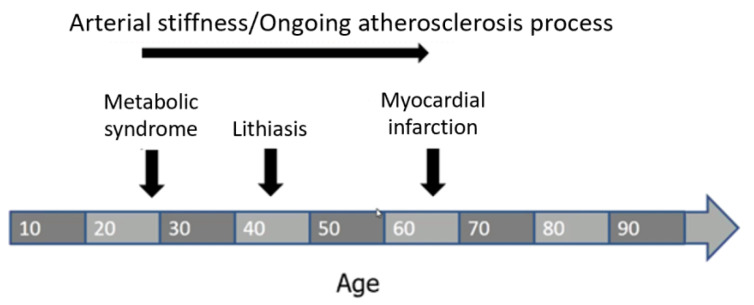
Temporal relationship between MS, stone episodes and CV events.

We can imagine that episodes of renal stones may occur in the long term, from the onset of metabolic derangements/MS to atherosclerosis and in CV morbidities (Figure 2).

These episodes might be considered a warning sign of an ongoing silent atherosclerotic process, with which it ultimately shares the same aetiology.

Therefore, patients with nephrolithiasis need to be exhaustively investigated, not only regarding the stone disease, urinary tract complications, and prevention of recurrences, but also regarding the systemic conditions for which the stone patients are at risk: renal failure, bone metabolic disease, and CV disease. This concept has been adopted by the recent guidelines for urolithiasis of the European Association of Urology [125].

The evaluation of cardiometabolic risk factors and MS components (blood pressure, cholesterol, glucose, BMI, smoking, etc.) should be routine in the assessment of renal stone formers. This would allow for treatment with either dietary and lifestyle interventions or, in more severe cases, with drugs. Regarding diet and lifestyle, the Mediterranean diet and the Dietary Approaches to Stop Hypertension (DASH) diet, whose beneficial effects on CV risk are well known, have been shown to decrease the incidence of nephrolithiasis [126,127].

In conclusion, we believe that a stone episode should be interpreted as a convenient ‘red flag’ to investigate and modify the patient’s cardiometabolic profile before the development of a CV complication—an event that is much more severe for the patient and expensive for public health.

## Data Availability

Not applicable.
